# PFOA biomonitoring and kidney cancer risk: a meta-analysis of serum levels

**DOI:** 10.3389/fonc.2025.1593300

**Published:** 2025-07-30

**Authors:** Francesca Spyrakis, Gioele Antonio Tiburtini, Stefania Bruno, Tommaso A. Dragani, Francesca Colombo

**Affiliations:** 1 Department of Drug Science and Technology, University of Turin, Turin, Italy; 2 Department of Medical Sciences, University of Turin, Turin, Italy; 3 Molecular Biotechnology Center, University of Turin, Turin, Italy; 4 Department of R&D, Aspidia srl, Milan, Italy; 5 Institute for Biomedical Technologies, National Research Council, Segrate, Italy

**Keywords:** biomonitoring, kidney, PFAS, PFOA, renal cancer

## Abstract

**Introduction:**

The potential link between perfluorooctanoic acid (PFOA) exposure and kidney cancer risk in humans remains uncertain. This meta-analysis aims to clarify the association by analyzing serum PFOA levels, a direct biomarker of internal exposure, rather than relying on indirect environmental or occupational measures.

**Methods:**

A systematic literature search was conducted using PubMed and Web of Science to identify relevant studies. Random-effects models were applied to pool effect estimates for both continuous serum PFOA levels and categorical comparisons (highest vs. lowest exposure groups). Subgroup and sex-stratified analyses were also performed.

**Results:**

Three studies met the inclusion criteria, encompassing 1,011 kidney cancer cases and 2,251 controls. Analysis of continuous PFOA levels yielded a non-significant meta-relative risk (mRR) of 1.05 (95% CI: 0.69–1.60), with substantial heterogeneity. The highest versus lowest exposure comparison also showed no significant association (mRR: 0.98; 95% CI: 0.64–1.50). Sex-stratified results from two studies revealed no significant differences in risk.

**Discussion:**

The findings suggest that any increased kidney cancer risk related to serum PFOA exposure is likely small and not statistically significant based on current evidence. Despite biological plausibility for renal toxicity, epidemiological data remain inconclusive. Further research with larger populations and standardized exposure assessment is needed to determine PFOA’s potential carcinogenic effects on the kidney.

## Introduction

Perfluorooctanoic acid (PFOA) is a synthetic fluorocarboxylic acid that belongs to the chemical family of per- and polyfluoroalkyl substances (PFAS), a very heterogeneous family that includes more than 12,000 different substances (1). It has been used as a surfactant in emulsion polymerization to produce fluorinated polymers and in many other applications, including firefighting foams, cosmetic formulations, textiles, etc. (2). Because a growing body of evidence has shown that PFOA is bioaccumulative, highly persistent, toxic, and ubiquitous in the environment and in humans (3), in Europe, under the Stockholm Convention, PFOA has been banned under the Persistent Organic Pollutants (POPs) Regulation as of July 4, 2020 (4).

PFOA was recently classified as a Group 1 human carcinogen by the International Agency for Research on Cancer (IARC) in Lyon, France. The Group 1 classification for PFOA was based on the combination of (i) limited evidence of human carcinogenicity for renal cancer and testicular cancer, (ii) sufficient evidence of carcinogenicity in laboratory animals, and (iii) strong mechanistic evidence of epigenetic alterations and immunosuppression in exposed humans (5).

Without challenging the IARC classification or the well-documented carcinogenicity of PFOA in animal models, our objective is to critically assess the human epidemiological evidence linking PFOA exposure to renal cancer, with a particular focus on the biological plausibility of this association. A key element in evaluating this risk lies in accurately quantifying human exposure to PFOA. In this context, serum levels of PFOA are widely recognized as a reliable indicator of cumulative exposure, regardless of the exposure pathway. Human biomonitoring plays a crucial role in this process, as it directly measures the internal dose of hazardous chemicals within the body. Unlike environmental monitoring, which only estimates potential external exposures (e.g., in air, water, or soil), biomonitoring provides an integrated assessment of actual internal exposure from all sources, including food, water, air, and consumer products (6, 7). Therefore, it serves as a fundamental tool for assessing the relationship between PFOA body burden and adverse health outcomes such as renal cancer.

PFOA is characterized by a long biological half-life, estimated to range from 1.48 to 5.1 years (8). This prolonged retention in the body indicates that serum PFOA levels primarily reflect cumulative exposure over an extended period rather than recent contact with contaminated sources. Consequently, due to PFOA’s bioaccumulation potential, blood levels serve as a robust biomarker of cumulative exposure, providing a reliable measure of long-term body burden rather than transient or estimated workplace exposures (9). This cumulative representation is particularly crucial for assessing potential health risks, including the development of kidney cancer.

Several studies have examined the risk of kidney cancer associated with exposure to PFOA. Some of these studies have reported statistically significant associations between PFOA exposure levels and an increased risk of kidney cancer (10–12) while others have not confirmed these findings (13–17). However, by histologic subtypes analysis, 17, reported a statistically significant association between serum PFOA concentrations and risk of renal cell carcinoma of the kidney among women [hazard ratio (HR) and 95% confidence interval (CI) per PFOA doubling: 1.54 (95% CI: 1.05, 2.26)] but not men (17).

The potential mechanism of PFOA in renal carcinogenesis remains unclear. It is uncertain whether the established mechanisms of PFOA action, primarily mediated through binding to peroxisome proliferator-activated receptor alpha (PPARα) in the liver, also play a role in the kidney (18, 19).

In this study, we performed a meta-analysis to assess the association between serum PFOA levels and kidney cancer risk. Unlike exposure estimates, which have inherent limitations, biomonitoring of PFOA blood levels provides a more accurate measure of internal exposure (6). Additionally, we explored the biological plausibility of a kidney-specific relationship between elevated serum PFOA levels and an increased risk of kidney cancer.

## Methods

The meta-analysis took into account the recommendations of the Preferred Reporting Items for Systematic Reviews and Meta-Analyses (PRISMA) (20). The literature search was conducted independently by two researchers (TAD, FC) using PubMed and Web of Science databases for articles published up to February 25, 2025. In our search, we used a combination of keywords synonymous with PFOA blood levels and kidney cancer risk. Publications were independently reviewed and selected by two authors (FC and TAD) for inclusion.

Meta-analysis was performed in R environment, using the metagen function of the meta package. In detail, we used log-transformed precalculated effect size measures and their standard errors (calculated from the 95% confidence intervals reported in the studies). A secondary analysis of the effects of the fourth quartile (fifth quartile in one study) versus the first quartile was performed using a similar case-control method, with the control being the first quartile of PFOA exposure. Forest and funnel plots were drawn using the meta::forest and meta::funnel functions, respectively. Random effect models were tested. The *I*^2^ statistic was used to calculate the between-studies heterogeneity. Publication bias was measured by performing Egger’s test with the metabias function (with k.min = 3) of meta package.

## Results

**Figure 1:**
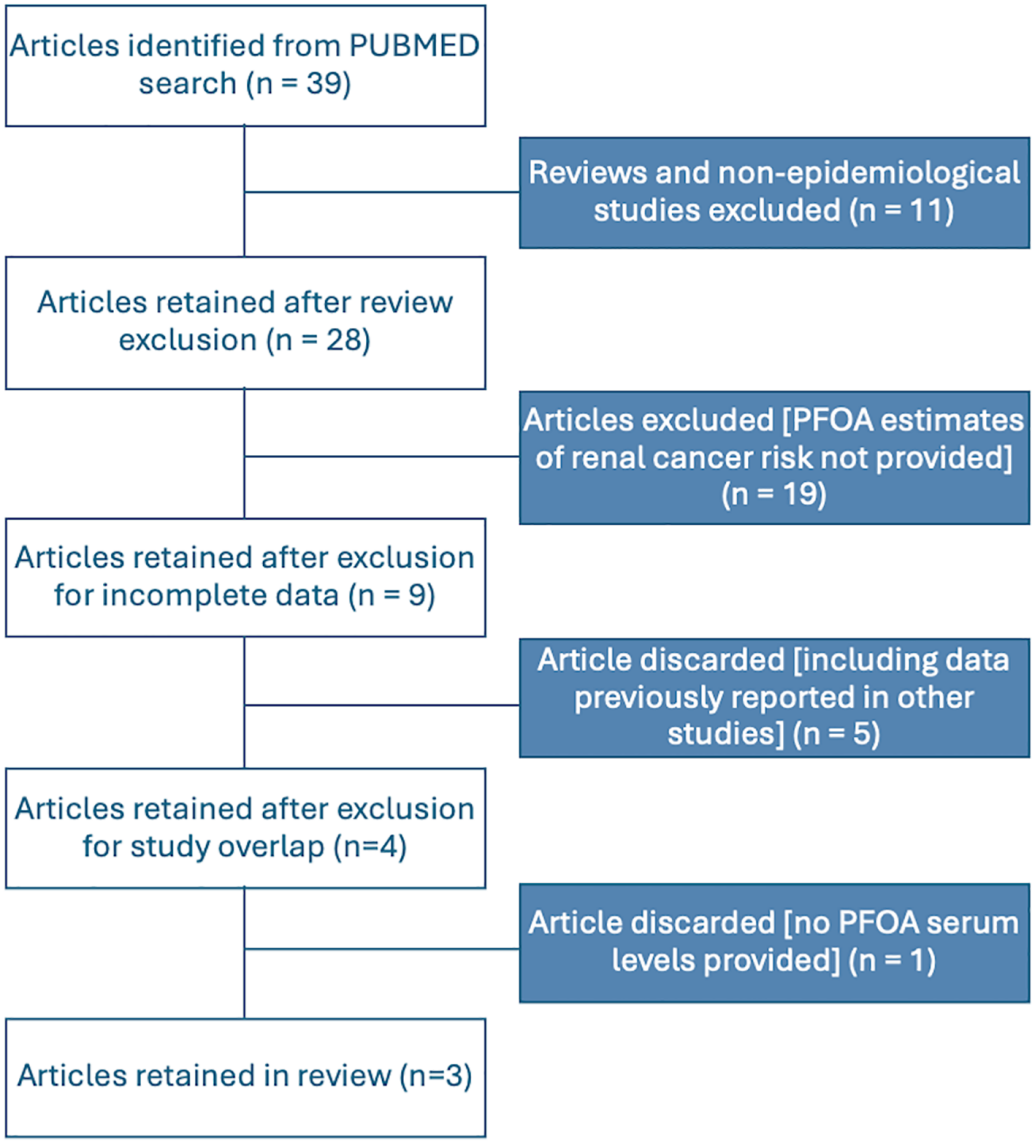
Flowchart for the identification and selection of studies to be included in the meta-analyses.

Based on our comprehensive literature search and study selection process, we included three independent studies in the meta-analysis. All the selected studies met the criteria for reporting PFOA serum levels. **Figure 1** illustrates the flow chart detailing our scientific literature search and study selection process, leading us to use three articles in our meta-analysis. Further details of the included studies and their references are provided in **Table 1**.

**Table 1:** Characteristics of the studies included in the meta-analysis.

First author and year	Reference	Study design	Exposure source	Potential confounders included in the analysis	Data availability for sex-stratified meta-analyses
Steenland et al., 2022	(12)	Cohort	Occupational	Exposure, hypertension, BMI	No
Rhee et al., 2023	(16)	Nested case-control	Environmental	BMI, estimated glomerular filtration rate, smoking status, hypertension history	Yes
Winquist et al., 2024	(17)	Case–cohort	Environmental	year of serum sample collection, age at serum sample collection, BMI, race, education, smoking status and alcohol consumption	Yes

Regarding study selection, we excluded data from (13, 14), and (11) from our analysis, as these studies were incorporated into (12), which was included in our meta-analysis. The study by (10) was also excluded due to its partial overlap with the population in (14) and its limitations in assigning historical estimated serum levels. Additionally, it had a limited number of cases with exposure estimates based on residence at the time of diagnosis and used cancer registry cases as controls, excluding several cancer types. Lastly, the occupational study by (15) was not included in our meta-analysis, as it lacks estimates of individual serum levels.

**Figure 2:**
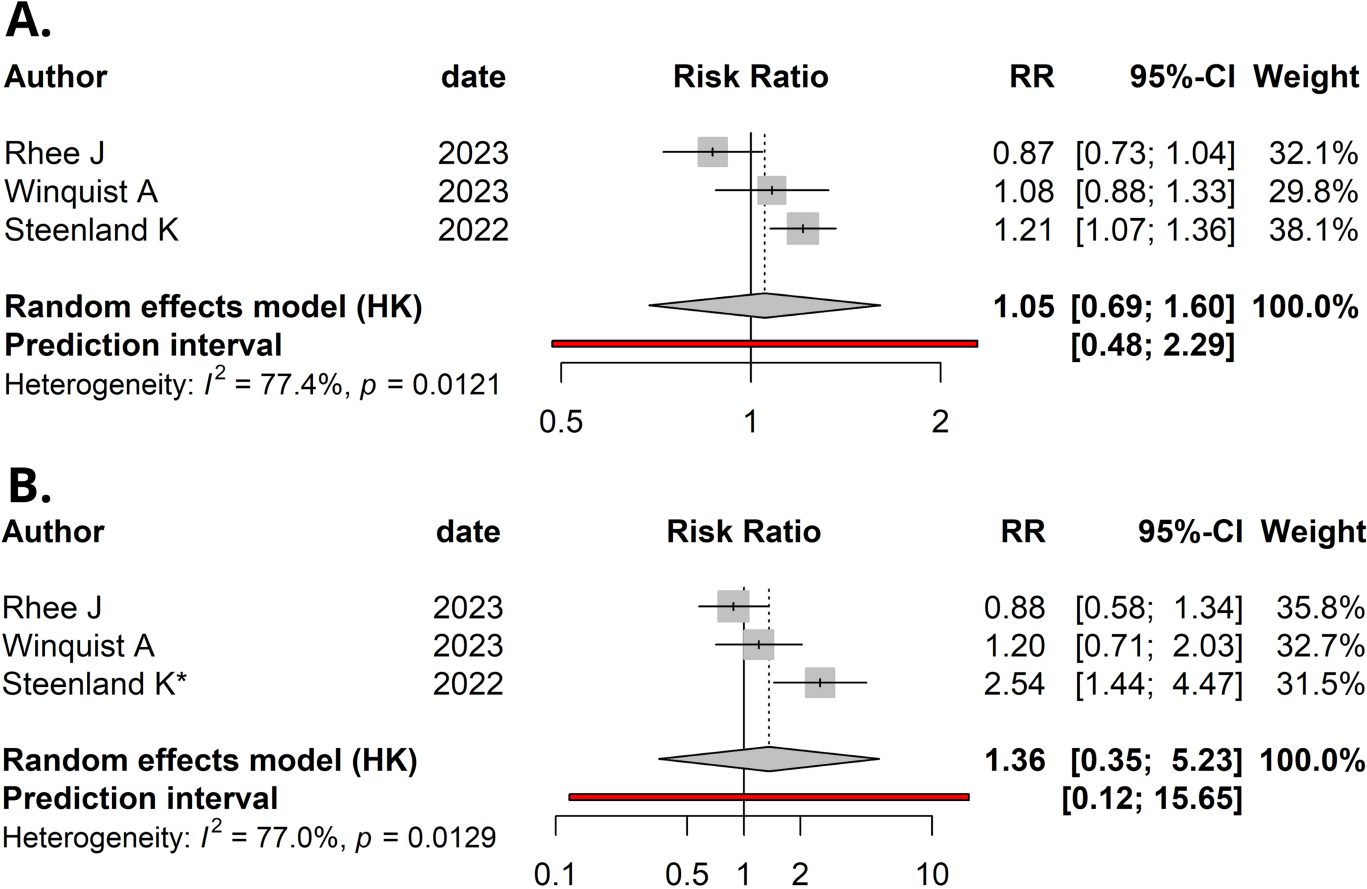
Forest plot (random-effects model) of studies’ relative risks, 95% confidence intervals (CI), and meta-analyses for: **(A)** Per natural log-unit increase in serum/plasma PFOA concentrations (ng/mL) and renal cancer risk. **(B)** Upper versus lower quartile in serum/plasma PFOA concentrations and renal cancer risk. *, Upper quintile data was used, as quartiles were not available in Steenland et al. *I*^2^, Higgins & Thompson’s statistic.

**Figure 3:**
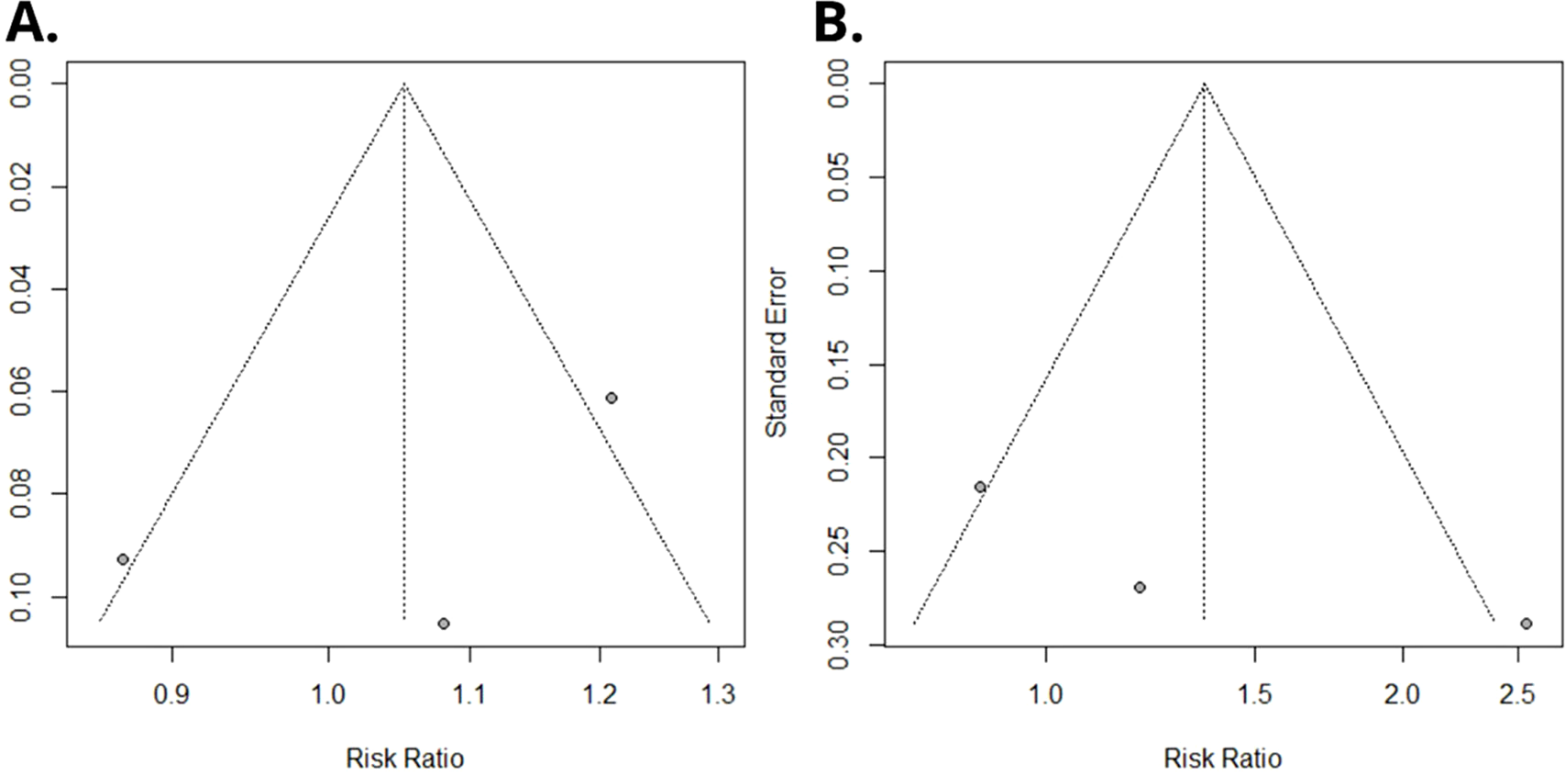
Funnel plot of Egger’s test on the associations between PFOA exposure and risk of renal cancer among studies included in the meta-analysis. **(A)** Overall serum/plasma PFOA concentrations (ng/mL) and renal cancer risk. **(B)** Upper versus lower quartile in serum/plasma PFOA concentrations and renal cancer risk.

Using a random-effects model, the meta-analysis of relative risks for kidney cancer, based on total quantitative exposure data, and including 1,011 cases and 2,251 controls, yielded a meta-relative risk (mRR) of 1.05 (95% CI: 0.69–1.60), with statistically significant heterogeneity among the studies (*I²* = 77.4%, P = 0.012) (**Figure 2A**). The meta-analysis comparing the highest exposure quartile to the lowest quartile (with 12 reporting quintiles, which were incorporated into our analysis) produced an mRR of 1.36 (95% CI: 0.35–5.23) (**Figure 2B**), with statistically significant heterogeneity among the studies (*I²* = 77.0%, P = 0.013). No publication bias was detected in either meta-analysis (P = 0.49 and P = 0.30, respectively). Funnel plots are presented in **Figure 3**.

In addition, we performed meta-analysis stratified by sex, with data available from just two studies. In both sexes, the mRRs were not statistically significant (mRR_males_ = 0.95, 95% CI: 0.34 – 2.64; mRR_females_ = 0.98, 95% CI: 0.01 – 74.52; **Supplementary Figure 1**). We found significant heterogeneity between studies, only in the meta-analysis with data from females (*I*^2males^ = 0%, *P*_males_ = 0.50 and *I*^2females^ = 79%, *P*_females_ = 0.03). Calculation of the publication bias was not possible with just two studies included in the meta-analyses stratified by sex.

We repeated the analyses using data restricted to the renal cell carcinoma (RCC) histotype. Indeed, Winquist et al. also reported results from the analysis on individuals who developed this specific histotype of kidney cancer (the other studies already focused on RCC). The results of these meta-analysis, that included 962 cases and the same number of controls as above, were similar to those already shown: on the basis of quantitative exposure data, we observed a mRR of 1.05 (95% CI: 0.68–1.60), with statistically significant heterogeneity among the studies (*I²* = 77.5%, *P* = 0.012; **Supplementary Figure 2A**). The meta-analysis comparing the highest exposure quartile to the lowest quartile produced an mRR of 1.37 (95% CI: 0.35–5.35) (**Supplementary Figure 2B**), with statistically significant heterogeneity among the studies (*I²* = 76.9%, P = 0.013). No publication bias was detected in either meta-analysis (P = 0.55, both). Funnel plots are presented in **Supplementary Figures 2C, D**. When we did sex-stratified meta-analyses (using the two studies with these data available), however, we did not confirm the association between serum PFOA concentrations and risk of renal cell carcinoma of the kidney among women reported by Winquist et al. (**Supplementary Figure 3**).

Since in two of the three studies (Steenland et al. and Winquist et al.) the majority of patients included were white, and the study of Rhee et al. reported also the results of the analysis stratified by ethnicity, we performed an additional meta-analysis considering this subset of individuals (based on total quantitative exposure data and including a total of 663 cases and 1,903 controls). We observed an mRR = 1.19 (95% CI: 0.92–1.53), with no significant heterogeneity among the studies (*I²* = 20.8%, P = 0.28). No publication bias was detected (P = 0.66). Results are shown in **Supplementary Figure 4**.

Finally, we carried out an additional meta-analysis with the subset of subjects whose blood samples were collected before 2002. Since the pooled analysis of Steenland et al. included data from the cohort analyzed by (11), recruited before 2002, and by (14), whose participants had blood withdrawal in 2005-2006, we decided to perform the meta-analysis using the data from Shearer et al., together with those from Winquist et al. (all before 2002) and the subgroup before 2002 in Rhee et al. including a total of 571 cases and 1,403 controls. Again, no significant association was observed between PFOA serum levels and kidney cancer risk (mRR = 1.35, 95% CI: 0.71–2.57; **Supplementary Figure 5**). The studies are quite heterogeneous (*I²* = 65%, *P* = 0.058) and there was no publication bias (*P* = 0.55).

## Discussion

Our meta-analysis found no statistically significant association between serum PFOA levels and renal cancer risk, regardless of whether exposure was analyzed as a continuous variable (reflecting quantitative levels) or as a categorical variable comparing the highest and lowest exposure groups. The analysis based on quantitative exposure showed significant heterogeneity, whereas the high versus low exposure comparison did not. Similarly, sex-stratified meta-analyses revealed no statistically significant associations in either males or females.

Two other meta-analyses showed results somehow comparable with ours. Bartell and Vieira (21) reported a statistically significant increase in kidney cancer risk per 10 ng/mL increase in serum PFOA levels (RR = 1.16; 95% CI: 1.03–1.30), but a non-significant increase per log_e_ increase in serum PFOA (RR = 1.49; 95% CI: 0.77–2.88). However, their meta-analysis included studies that did not report serum PFOA levels, requiring the authors to estimate these values. As a result, the calculated meta-risks do not strictly reflect real PFOA blood levels but rather an approximation of PFOA exposure (21).

The meta-analysis by Seyyedsalehi and Boffetta (22) reported a statistically significant association between overall PFAS exposure and kidney cancer risk (RR = 1.18; 95% CI: 1.05–1.32; *I²* = 52.8%, 11 studies). However, this analysis included studies assessing exposure to PFAS mixtures as well as studies without data on PFOA blood levels, limiting its ability to specifically assess the risk associated with PFOA blood levels (22).

Thus, our meta-analysis is the first to estimate the meta-risk of kidney cancer in relation only to blood PFOA levels, providing a direct measure of actual exposure. However, we acknowledge its limited statistical power, as only three studies met the inclusion criteria. Notably, the study by (12), included in our analysis, comprised a pooled analysis of a large dataset of kidney cancer cases and controls, enhancing the robustness of our findings.

A limitation of our results and those of other studies on this topic is the multicollinearity between exposure to PFOA and other PFAS; in reality, exposure to a single PFAS is rare; it almost always involves mixtures of different PFAS, the composition of which is often not fully known (23). Multicollinearity in the levels of exposure to different PFASs complicates the interpretation of the results of epidemiological studies and renders the estimates of the statistical coefficients related to a specific PFAS under investigation partially unreliable. This problem cannot be solved, but it can be mitigated by increasing the size of studies, improving the precision of measurements of PFAS present in the blood of individuals, and validating the results obtained in independent studies.

Another limitation is the variability of serum PFOA levels over time (24) and their potential relevance to cancer risk. A long-term prospective epidemiologic study monitoring serum PFAS levels and their association with cancer risk over several decades would be necessary to fully address this issue. While our results showed no statistically significant association between PFOA serum levels and an increased risk of kidney cancer, we believe that further studies are needed to explore this important question more comprehensively.

Regardless of the statistical association between high PFOA blood levels and an increased risk of kidney cancer, is there biological plausibility for such a link in humans? Carcinogenicity studies in rodents, which are often used to establish biological plausibility and help interpret uncertain epidemiologic findings (25), have not provided evidence supporting PFOA-induced kidney carcinogenicity. A long-term study in male and female rats exposed to ammonium pentadecafluorooctanoate at 30 ppm or 300 ppm (approximately 1.5 and 15 mg/kg) found no increase in kidney tumor incidence, though a significant rise in testicular Leydig cell tumors was observed in male rats (26). More recently, a long-term carcinogenicity study assessing both perinatal and postnatal PFOA exposure reported increased incidences of hepatocellular and pancreatic neoplasms in male rats, as well as pancreatic tumors in female rats, but no increase in kidney tumor incidence in either sex (27).

However, laboratory rodent models may not accurately reflect the carcinogenic potential of PFOA and other PFAS in humans due to substantial differences in their elimination half-lives. In humans, the half-life of PFOA is approximately four years, whereas in rodents, it ranges from just a few days to hours. This significant discrepancy in toxicokinetics may limit the relevance of rodent studies for assessing long-term human health risks (8, 28–30).

Renal PFOA toxicity might contribute to kidney cancer risk. Elevated serum PFOA concentrations have been linked to decreased estimated glomerular filtration rate (eGFR), a key indicator of kidney damage (31). Additionally, studies have reported associations between high serum PFOA levels and hyperuricemia, a biomarker linked to hypertension, diabetes mellitus, cardiovascular disease, inflammation, and chronic kidney disease (32–34).

However, a large longitudinal study (n = 32,254) on chronic kidney disease in adults from a Mid-Ohio Valley community exposed to elevated PFOA levels through contaminated drinking water found no statistically significant association between PFOA exposure and chronic kidney disease (35). These findings challenge the hypothesis of an association between high serum levels of PFOA and renal toxicity.

We should also consider the possibility of reverse causation, as impaired renal function, such as in chronic kidney disease (CKD), can reduce the clearance of PFOA, potentially leading to higher serum levels. This raises the concern that elevated PFOA concentrations observed in some studies may be a consequence, rather than a cause, of kidney dysfunction.

## Conclusions

The current epidemiological evidence on the carcinogenicity of PFOA in the human kidney remains limited and inconclusive. While some studies have reported a statistically significant association between elevated serum PFOA levels and increased kidney cancer risk, others have failed to replicate these findings. Our meta-analysis does not support a statistically significant association between serum PFOA levels and kidney cancer risk, suggesting that the existing human data are insufficient to establish a causal relationship.

Mechanistic evidence is similarly inconclusive. Although PFOA has demonstrated carcinogenic potential in rodent models, it has not been shown to induce renal tumors in these species. Importantly, the substantial species differences in PFOA toxicokinetics, particularly the much shorter half-life in rodents, limit the translational relevance of these findings to humans.

Chronic inflammation has been proposed as a plausible mechanistic pathway linking PFOA exposure to renal carcinogenesis, but current experimental evidence remains too sparse and inconsistent to confirm this hypothesis.

In conclusion, while a potential link between high PFOA exposure and kidney cancer risk cannot be entirely ruled out, the current body of evidence does not provide robust epidemiological or mechanistic support for this association. There is a clear need for well-designed prospective studies with accurate exposure assessment and mechanistic investigations using models that better reflect human toxicodynamics. These efforts are essential to clarify whether PFOA contributes to renal carcinogenesis and to inform evidence-based regulatory and public health decisions.

## Data Availability

The original contributions presented in the study are included in the article/**Supplementary Material**. Further inquiries can be directed to the corresponding author.

## Supplementary material

Supplementary Figure 1 Forest plot (random-effects model) of studies’ relative risks, 95% confidence intervals (CI), and meta-analyses per natural log-unit increase in serum/plasma PFOA concentrations (ng/mL) and renal cancer risk in **(A)** females and **(C)** males. Funnel plot of Egger’s test on the associations between PFOA exposure and risk of renal cancer among studies included in the meta-analysis in females **(B)** and males **(D)**.

Supplementary Figure 2 Forest plot (random-effects model) of studies’ relative risks, 95% confidence intervals (CI), and meta-analyses for: **(A)**. Per natural log-unit increase in serum/plasma PFOA concentrations (ng/mL) and RCC risk. **(B)**. Upper versus lower quartile in serum/plasma PFOA concentrations and RCC risk. *, Upper quintile data was used, as quartiles were not available in Steenland et al. § Only RCC data were used from the study by Winquist et al. *I*^2^, Higgins & Thompson’s statistic. Funnel plot of Egger’s test on the associations between PFOA exposure and risk of RCC among studies included in the meta-analysis with continuous data **(C)** and quartiles **(D)**.

Supplementary Figure 3 Forest plot (random-effects model) of studies’ relative risks, 95% confidence intervals (CI), and meta-analyses per natural log-unit increase in serum/plasma PFOA concentrations (ng/mL) and RCC risk in **(A)** females and **(B)** males. Funnel plot of Egger’s test on the associations between PFOA exposure and risk of RCC among studies included in the meta-analysis in females **(C)** and males **(D)**.

Supplementary Figure 4 **(A)** Forest plot (random-effects model) of studies’ relative risks, 95% confidence intervals (CI), and meta-analyses for per natural log-unit increase in serum/plasma PFOA concentrations (ng/mL) and kidney cancer risk in Whites. * Only RCC data were used from the study by Rhee et al. *I*^2^, Higgins & Thompson’s statistic. **(B)** Funnel plot of Egger’s test on the associations between PFOA exposure and risk of kidney cancer in Whites among studies included in the meta-analysis.

Supplementary Figure 5 **(A)** Forest plot (random-effects model) of studies’ relative risks, 95% confidence intervals (CI), and meta-analyses for per natural log-unit increase in serum/plasma PFOA concentrations (ng/mL) and kidney cancer risk in subjects whose blood sample collection was done before 2002. * Only data before 2002 were used from the study by Rhee et al. *I*^2^, Higgins & Thompson’s statistic. **(B)** Funnel plot of Egger’s test on the associations between PFOA exposure before 2002 and risk of kidney cancer among studies included in the meta-analysis.
