# Correction: PFOA biomonitoring and kidney cancer risk: a meta-analysis of serum levels

**DOI:** 10.3389/fonc.2025.1705495

**Published:** 2025-11-07

**Authors:** Francesca Spyrakis, Gioele Antonio Tiburtini, Stefania Bruno, Tommaso A. Dragani, Francesca Colombo

**Affiliations:** 1 Department of Drug Science and Technology, University of Turin, Turin, Italy; 2 Department of Medical Sciences, University of Turin, Turin, Italy; 3 Molecular Biotechnology Center, University of Turin, Turin, Italy; 4 Department of R&D, Aspidia srl, Milan, Italy; 5 Institute for Biomedical Technologies, National Research Council, Segrate, Italy

**Keywords:** biomonitoring, kidney, PFAS, PFOA, renal cancer

There was a mistake in **Figures 2** and **3**, as well as **Supplementary Figures 2** and **4** as published. In the original version of the article, we mistakenly used the beta coefficient for the association between log-PFOA and kidney cancer from Steenland et al., 2022, instead of its OR value.

**Figure 2:**
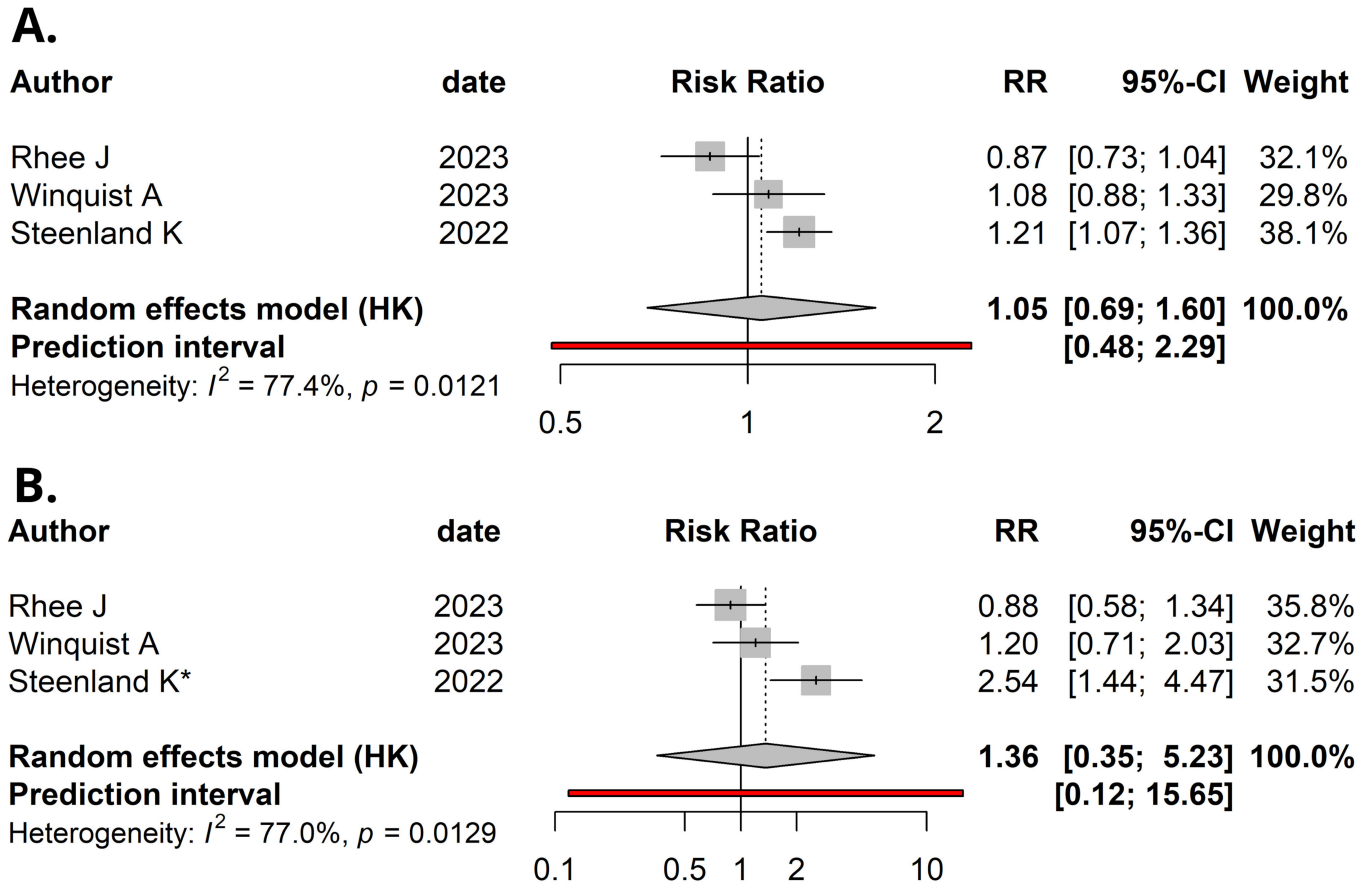
Forest plot (random-effects model) of studies’ relative risks, 95% confidence intervals (CI), and meta-analyses for: **(A)** Per natural log-unit increase in serum/plasma PFOA concentrations (ng/mL) and renal cancer risk. **(B)** Upper versus lower quartile in serum/plasma PFOA concentrations and renal cancer risk. *, Upper quintile data was used, as quartiles were not available in Steenland et al. *I*², Higgins & Thompson’s statistic.

**Figure 3:**
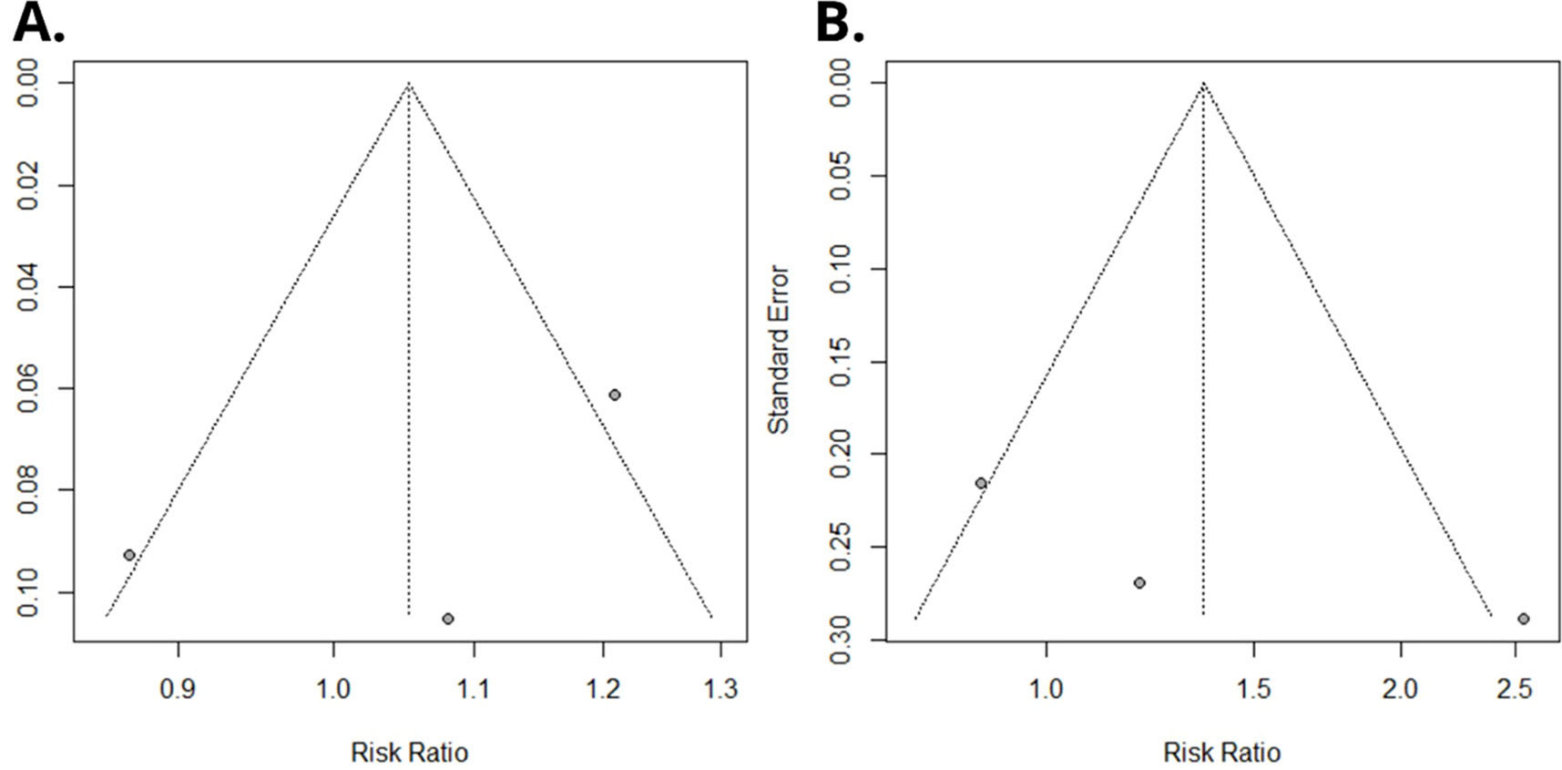
Funnel plot of Egger’s test on the associations between PFOA exposure and risk of renal cancer among studies included in the meta-analysis. **(A)** Overall serum/plasma PFOA concentrations (ng/mL) and renal cancer risk. **(B)** Upper versus lower quartile in serum/plasma PFOA concentrations and renal cancer risk.

The corrected **Figures 2** and **3** appear below. The correct **Supplementary Figures 2** and **4**, have now been replaced.

In the **Abstract**, “0.59 (95% CI: 0.06–5.89)” was wrong.

This has been corrected to read:

“1.05 (95% CI: 0.69–1.60)”

In the **Abstract**, 0.98 (95% CI: 0.64–1.50) was wrong.

This has been corrected to read:

“1.36 (95% CI: 0.35–5.23)”

“0.59 (95% CI: 0.06–5.89)” was an error.

“(*I*² = 90%, P < 0.01)” was an error.

A correction has been made to the section **Results**, paragraph 3:

“1.05 (95% CI: 0.69–1.60)”

“(*I*^2^ = 77.4%, P = 0.012)”

“0.98 (95% CI: 0.64–1.50)” was an error.

“with no statistically significant heterogeneity among the studies (*I*² = 0%, i = 0.66).” was an error.

A correction has been made to the section **Results**, paragraph 3:

“1.36 (95% CI: 0.35–5.23)”

“with statistically significant heterogeneity among the studies (*I*^2^ = 77.0%, P = 0.013)”

“(P = 0.30 and P = 0.81, respectively)” was an error.

A correction has been made to the section **Results**, paragraph 3:

“(P = 0.49 and P = 0.30, respectively)”

“0.59 (95% CI: 0.06–5.76)” was an error.

“(*I*² = 89%, P < 0.0001)” was an error.

A correction has been made to the section **Results**, paragraph 5:

“1.05 (95% CI: 0.68–1.60)”

“(*I*^2^ = 77.5%, P = 0.012)”

“0.96 (95% CI: 0.64–1.45)” was an error.

“with no statistically significant heterogeneity among the studies (*I*² = 0%, P = 0.71).” was an error.

“(P = 0.37 and P = 0.58, respectively)” was an error.

A correction has been made to the section **Results**, paragraph 5:

“1.37 (95% CI: 0.35–5.35)”

“with statistically significant heterogeneity among the studies (*I*² = 76.9%, P = 0.013).”

“(P = 0.55, both)”

“0.75 (95% CI: 0.04–16.01)” was an error.

“with a significant heterogeneity among the studies (*I*² = 91%, P < 0.01).” was an error.

“(P = 0.74)” was an error.

A correction has been made to the section **Results**, paragraph 6:

“1.19 (95% CI: 0.92–1.53)”

“with no significant heterogeneity among the studies (*I*² = 20.8%, P = 0.28).”

“(P = 0.66)”

The original version of this article has been updated.

