# Supplementary figures and images for: PFOA biomonitoring and kidney cancer risk: a meta-analysis of serum levels
Source: Front Oncol. 2025 Jul 30;15:1593300. doi: 10.3389/fonc.2025.1593300 (PMC12344524; doi:10.3389/fonc.2025.1593300)

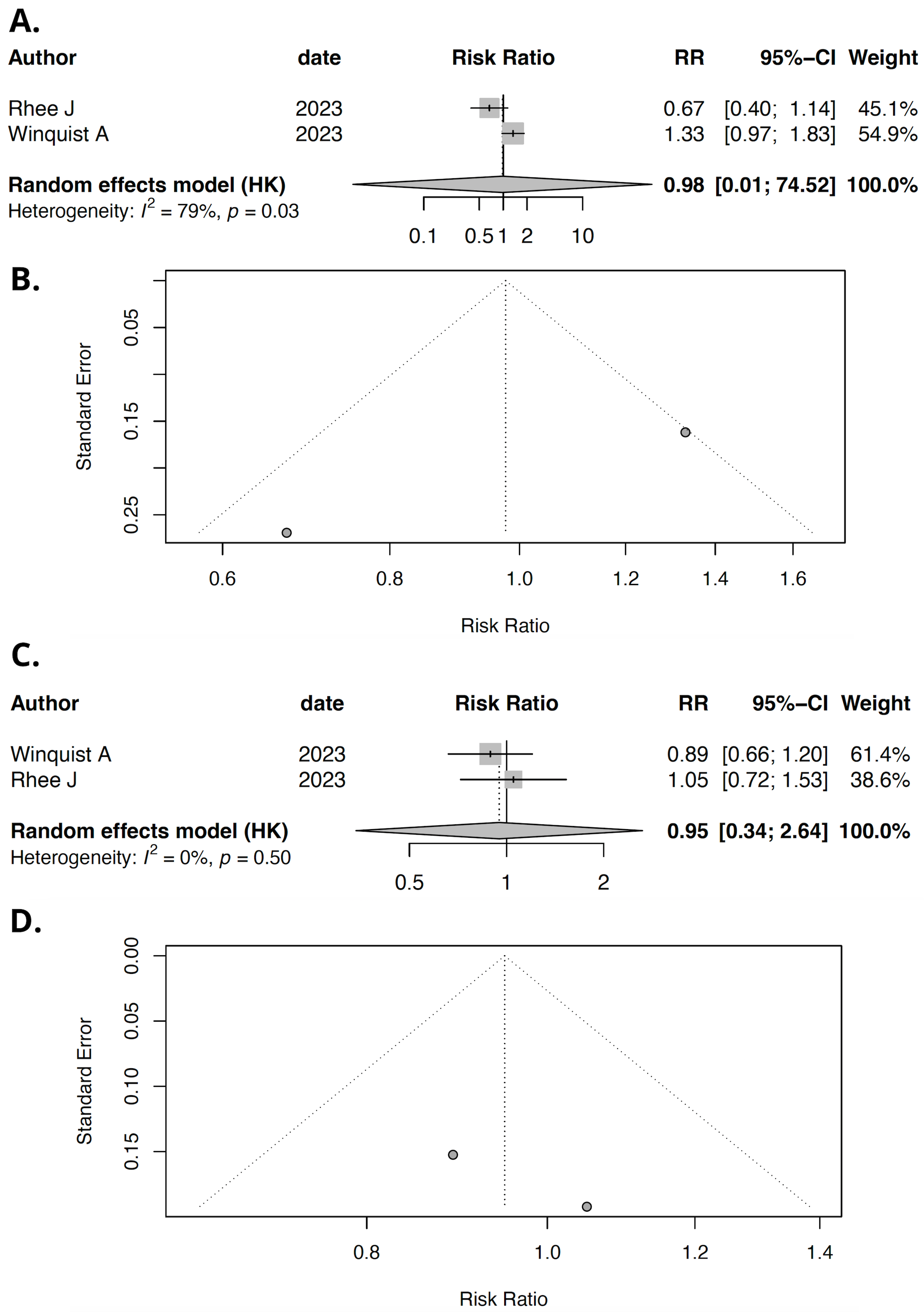

Supplement: Supplementary Figure 1 — Forest plot (random-effects model) of studies’ relative risks, 95% confidence intervals (CI), and meta-analyses per natural log-unit increase in serum/plasma PFOA concentrations (ng/mL) and renal cancer risk in (A) females and (C) males. Funnel plot of Egger’s test on the associations between PFOA exposure and risk of renal cancer among studies included in the meta-analysis in females (B) and males (D). [file Image1.tiff]

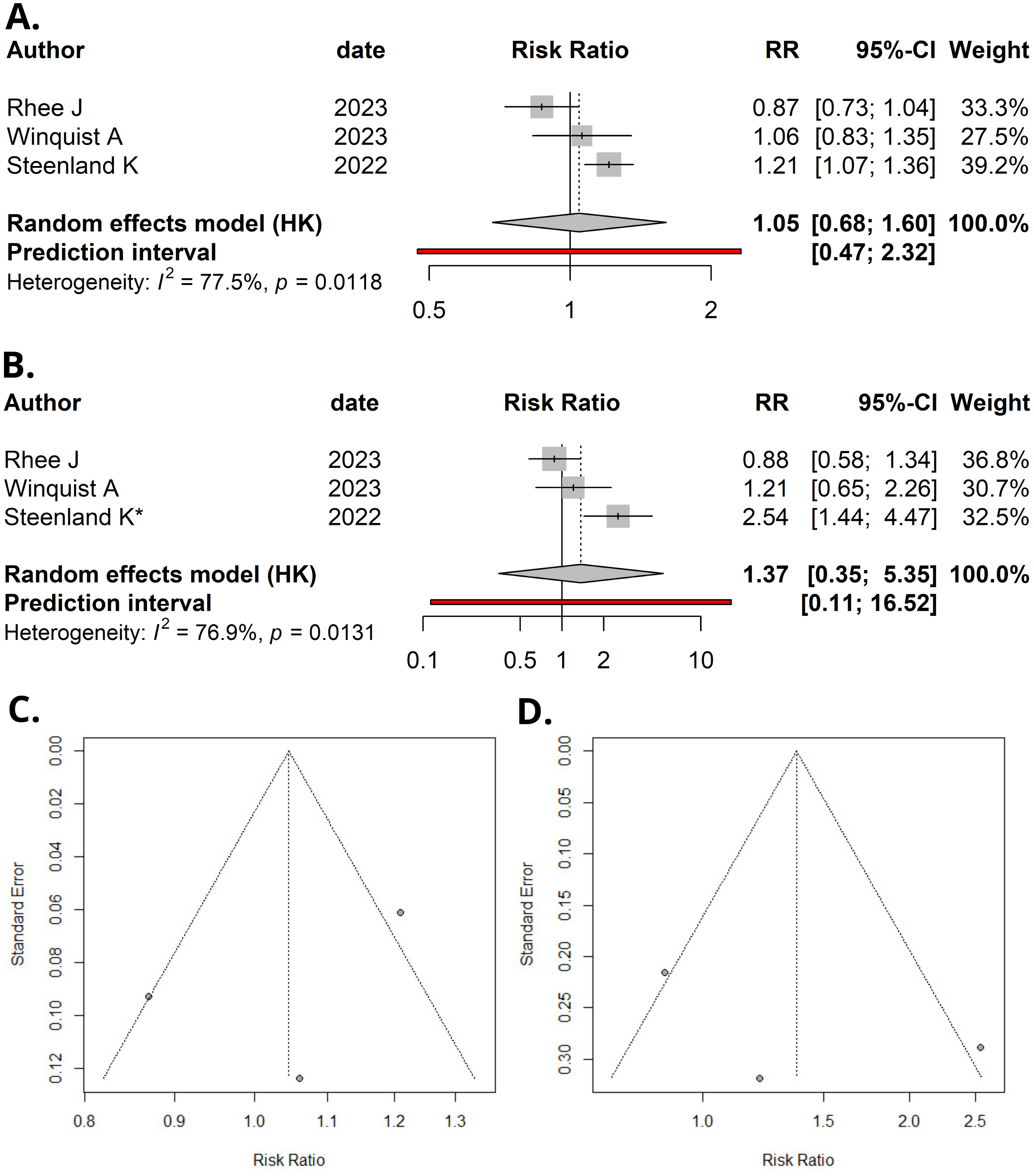

Supplement: Supplementary Figure 2 — Forest plot (random-effects model) of studies’ relative risks, 95% confidence intervals (CI), and meta-analyses for: (A). Per natural log-unit increase in serum/plasma PFOA concentrations (ng/mL) and RCC risk. (B). Upper versus lower quartile in serum/plasma PFOA concentrations and RCC risk. *, Upper quintile data was used, as quartiles were not available in Steenland et al. § Only RCC data were used from the study by Winquist et al. I2, Higgins & Thompson’s statistic. Funnel plot of Egger’s test on the associations between PFOA exposure and risk of RCC among studies included in the meta-analysis with continuous data (C) and quartiles (D). [file Image2.tiff]

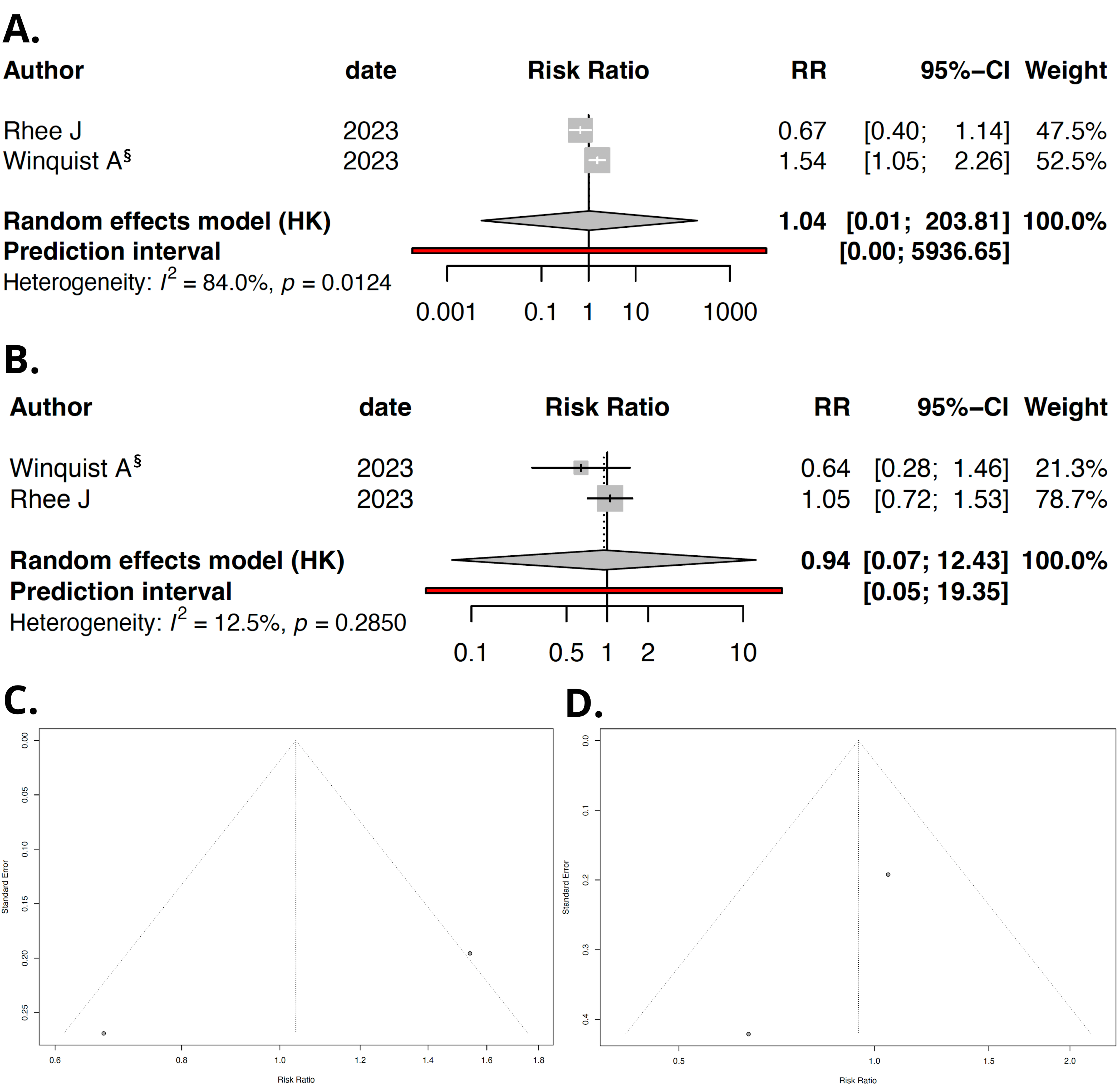

Supplement: Supplementary Figure 3 — Forest plot (random-effects model) of studies’ relative risks, 95% confidence intervals (CI), and meta-analyses per natural log-unit increase in serum/plasma PFOA concentrations (ng/mL) and RCC risk in (A) females and (B) males. Funnel plot of Egger’s test on the associations between PFOA exposure and risk of RCC among studies included in the meta-analysis in females (C) and males (D). [file Image3.tiff]

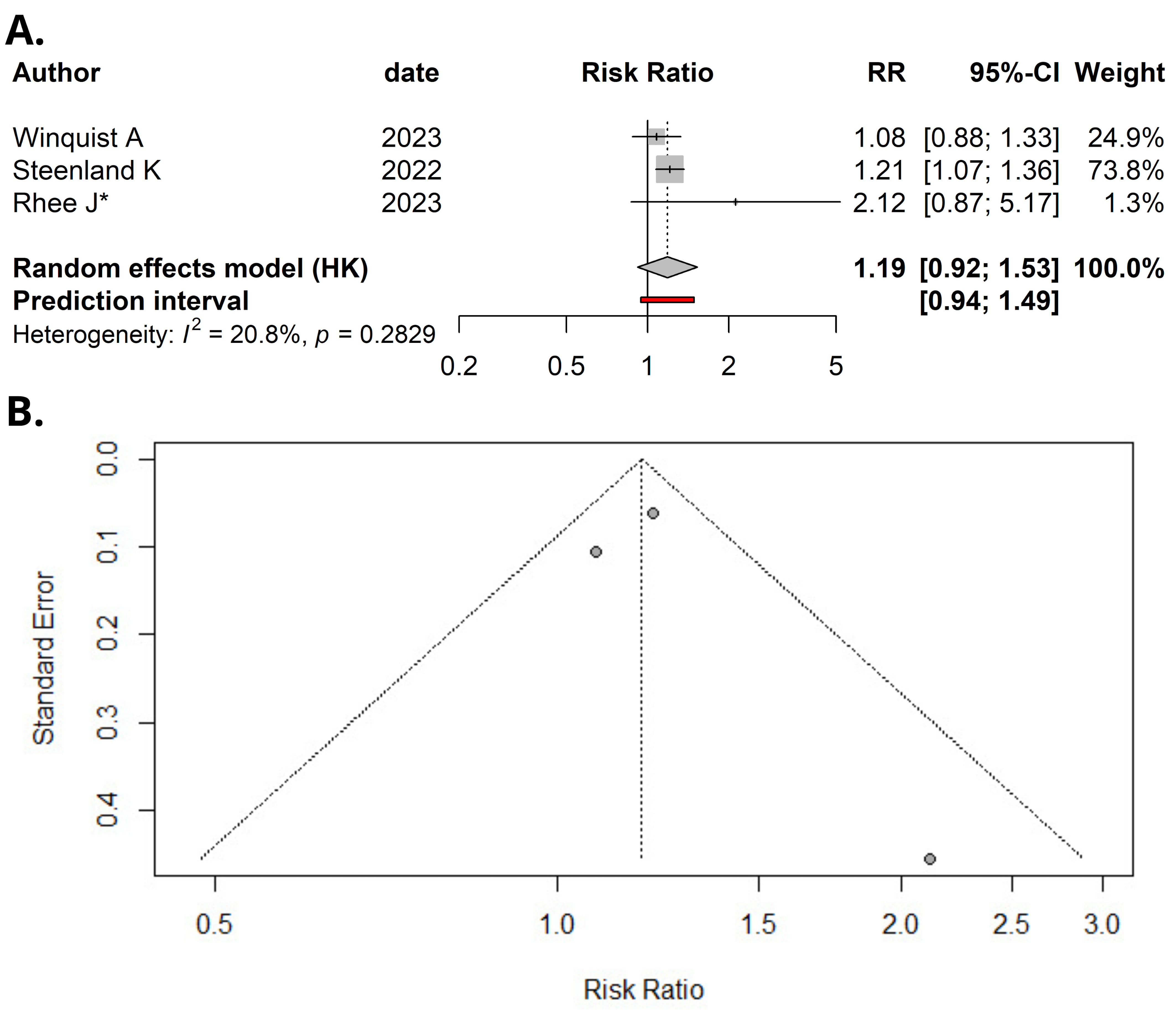

Supplement: Supplementary Figure 4 — (A) Forest plot (random-effects model) of studies’ relative risks, 95% confidence intervals (CI), and meta-analyses for per natural log-unit increase in serum/plasma PFOA concentrations (ng/mL) and kidney cancer risk in Whites. * Only RCC data were used from the study by Rhee et al. I2, Higgins & Thompson’s statistic. (B) Funnel plot of Egger’s test on the associations between PFOA exposure and risk of kidney cancer in Whites among studies included in the meta-analysis. [file Image4.tiff]

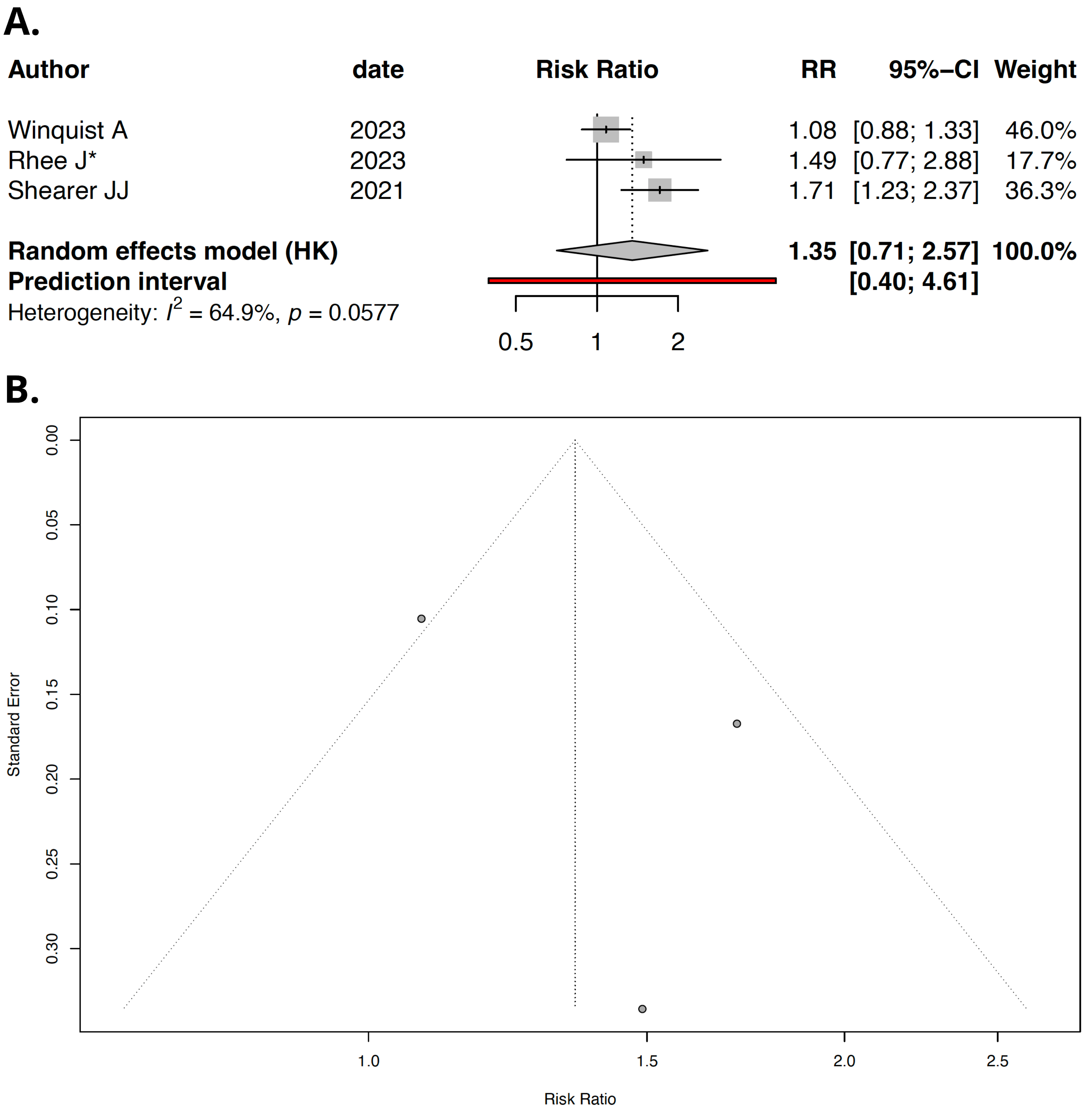

Supplement: Supplementary Figure 5 — (A) Forest plot (random-effects model) of studies’ relative risks, 95% confidence intervals (CI), and meta-analyses for per natural log-unit increase in serum/plasma PFOA concentrations (ng/mL) and kidney cancer risk in subjects whose blood sample collection was done before 2002. * Only data before 2002 were used from the study by Rhee et al. I2, Higgins & Thompson’s statistic. (B) Funnel plot of Egger’s test on the associations between PFOA exposure before 2002 and risk of kidney cancer among studies included in the meta-analysis. [file Image5.tiff]
